# The role of curriculum dose for the promotion of fruit and vegetable intake among adolescents: results from the Boost intervention

**DOI:** 10.1186/s12889-015-1840-0

**Published:** 2015-06-05

**Authors:** Thea Suldrup Jørgensen, Mette Rasmussen, Anne Kristine Aarestrup, Annette Kjær Ersbøll, Sanne Ellegaard Jørgensen, Elizabeth Goodman, Trine Pagh Pedersen, Pernille Due, Rikke Krølner

**Affiliations:** Centre for Intervention Research in Health Promotion and Disease Prevention, National Institute of Public Health, University of Southern Denmark, Øster Farimagsgade 5A, 2nd floor, Copenhagen, K 1353 Denmark; National Institute of Public Health, University of Southern Denmark, Øster Farimagsgade 5A, 2nd floor, Copenhagen, K 1353 Denmark; Division of General Academic Pediatrics, Massachusetts General Hospital for Children, Massachusetts General Hospital, 55 Fruit Street, Boston, MA 02114 USA

**Keywords:** Implementation, Curriculum dose, School, Adolescents, Fruit and vegetables

## Abstract

**Background:**

Multi-component interventions combining educational and environmental strategies have proved effective in increasing children and adolescents’ fruit and vegetable intake. However such interventions are complex and difficult to implement and several studies report poor implementation. There is a need for knowledge on the role of dose for behaviour change and for assessment of intervention dose to avoid conclusions that intervention components which are not implemented are ineffective. This study aimed to examine 1) the association between dose of a class curriculum and adolescents’ fruit and vegetable intake in a school-based multi-component intervention, 2) if gender and socioeconomic position modify this association.

**Methods:**

We carried out secondary analysis of data from intervention schools in the cluster-randomized Boost study targeting 13-year-olds’ fruit and vegetable intake. Teacher- and student data on curriculum dose delivered and received were aggregated to the school-level and class-level (only possible for student data). We analysed the association between curriculum dose and students’ (*n* 995) self-reported fruit and vegetable intake (24-h recall questionnaire) after finalization of the intervention using multi-level analyses. Potential moderation was examined by analyses stratified by gender and socioeconomic position.

**Results:**

Average dose received at class-level was significantly associated with students’ fruit and vegetable intake (10 g (CI: 0.06, 20.33) per curricular activity received). In stratified analyses the association remained significant among boys only (14 g (CI: 2.84, 26.76) per curricular activity received). The average dose delivered and received at the school-level was not significantly associated with students’ intake.

**Conclusions:**

We found a dose—response relationship between number of curricular activities received and adolescents’ fruit and vegetable intake. The results indicate that curriculum dose received only mattered for promotion of fruit and vegetable intake among boys. Future studies should explore this gender difference in larger samples to guide the planning of school-based curricular interventions with regards to the optimal number of curricular activities required to promote behavioural change in subgroups with low fruit and vegetable intake at baseline.

**Trial registration:**

Current Controlled Trials ISRCTN11666034.

## Background

Schoolchildren do not reach the international recommendations of eating at least 400 g of fruit and vegetables (FV) daily [1, 2]. School-based multi-component interventions combining educational and environmental strategies have proved effective in increasing children and adolescents’ FV intake [3–6]. However such interventions are complex and difficult to implement [7, 8] and several studies report poor implementation [9–12]. Important barriers for teachers’ implementation of curricular activities are duration of the intervention and time required for implementation, which calls for knowledge on the role of intervention dose for behaviour change for example the minimal dose required to achieve an effect [5, 13–15]. Furthermore an assessment of intervention dose delivered and received is important to avoid conclusions that intervention components which are not implemented are ineffective (type III error) [14, 16].

Previous studies have reported conflicting results on the association between dose delivered of dietary interventions and adolescents’ dietary behaviour change: The Pro Children Study and the Dutch Krachtvoer Healthy Diet Programme found a positive association between number of FV lessons delivered and changes in FV intake among 11-year-olds in Norway, Spain and the Netherlands [12] and 12–14-year-olds in the Netherlands [17], respectively. In contrast the Norwegian Fruits and Vegetables Make the Marks intervention found no relation between the dose of a home economics curriculum delivered in year six (mean age 11.3 years) and FV intake at first- and second follow-up [18]. There is a need for more research on the role of dose of curricular interventions targeting adolescents’ FV intake including the role of curriculum dose received reported by students.

While previous studies reported effect of curriculum dose on FV intake among all adolescents, little is known about the role of dose in subgroups with a low intake of FV such as boys and children of low socioeconomic position (SEP) [19–21]. Some studies show that health promoting interventions including educational strategies are not as effective among vulnerable groups such as low SEP and boys [22], whereas others suggest that subgroups with low baseline values respond better to FV interventions [21]. It is therefore relevant to study if the importance of curriculum dose for adolescents’ FV intake differ by subgroups.

The aim of this study was to examine *if dose of curricular activities delivered by teachers and received by students, respectively, was associated with intake of FV among 13-year-old students at the end of the intervention* in the school-based multi-component Boost intervention. We hypothesized a dose—response association with increasing FV intake by increasing curriculum dose. The second aim was to *investigate if the association differed by gender and SEP*. The study is a secondary analysis of the Boost intervention data and has been pre-specified in the trial registry Current Controlled Trials ISRCTN11666034 (http://www.isrctn.com/ISRCTN11666034).

## Methods

### The Boost study

The Boost intervention combined curricular activities and free FV distribution at school, parental newsletters and fact sheets for sports- and youth clubs to increase adolescents’ FV intake [23]. The intervention lasted nine months (September 2010 – May 2011). It was tested in a school-randomized controlled trial among all year seven students (≈13-year-olds) from a random sample of 20 intervention- and 20 control schools from 10 randomly selected municipalities in Denmark. Implementation of intervention components was monitored by a thorough quantitative and qualitative process evaluation [15, 24, 25].

### The Boost curriculum

The Boost curriculum was designed to change adolescents’ FV intake through changes in determinants such as knowledge, awareness, attitudes, taste preferences and influence from family, peers and media [19, 23, 26]. Curricular activities were developed specifically for Boost or based on existing material from other interventions including the Pro Children study and HEalth In Adolescents project [23]. The curricular activities were all related to FV and were designed to be integrated in different school subjects for example Danish, maths, geography, home economics and physical education and to meet national learning objectives for each subject [27]. At a one-day pre-intervention workshop, teachers acting as local Boost coordinators at each intervention school gave feedback on a preliminary version of the Boost curriculum to ensure local applicability. To ensure compliance with national learning objectives the teaching material was critically reviewed by primary school teachers who were part of a national Boost planning group and college teachers outside the project.

The Boost curriculum consisted of four main parts 1) a detailed teacher manual including 12 compulsory and 13 optional curricular activities, each to be carried out during 1–4 class lessons. A time schedule specified 1–3 activities which were to be implemented monthly to ensure regular delivery. The activities combined practical and theoretical approaches, such as analysing and creating advertisements related to FV/food, studying how FV affect the body, trying different tastes, and discussing social norms related to eating FV; 2) A teacher script for a project week to be conducted at school or in the school neighbourhood including four compulsory and four optional activities. The script included practical activities at school (for example, cookery) and activities in the school neighbourhood, such as field trips to local supermarkets, greengrocers, or fruit orchards; 3) A student workbook covering the activities presented in the teacher manuals and 4) a computer tailoring module which students were expected to complete three times. The Boost computer-tailored feedback messages were tailored to the students’ FV intake, awareness levels, taste preferences, and leisure time activities. Students’ answers were stored in the system, enabling them to monitor their own intake over time. Personal feedback generated by the computer module suggested recipes to try FV in a new way and contained ideas for eating FV with friends and at leisure time activities [23]. The teachers were to implement all compulsory activities in each of the year seven classes but were allowed to adapt them to their local context. The teaching material is available in Danish at www.cirhp.dk.

### Fruit and vegetable distribution and parental newsletters

Teachers were responsible for daily distribution of one piece of fruit or vegetable to students at class (free provision). To create a pleasant eating environment, teachers were encouraged to implement a FV break and to cut up the FV in appealing snacks. The Boost coordinators were asked to post six Boost parental newsletters at the school’s website for parents with ideas on how to increase adolescents’ FV intake at home and in their leisure time. The Boost intervention is described in details elsewhere [23].

### Study sample and data collection

We used baseline-and follow-up data from year seven students, parents, school principals, and teachers from the 20 intervention schools.

A total of 1175 students were enrolled at intervention schools. Before intervention start (August 2010), 1121 students completed a baseline questionnaire (response rate: 95.4 %) and of these 1060 students (90.7 %) completed a follow-up questionnaire at the end of the intervention (May/June 2011). The 24-h recall questionnaire used for estimation of total daily FV intake was completed by 1118 students at baseline and 1060 students at follow-up. As some students reported eating up to 4000 g of FV daily, we defined 1200 g as cut-point for the highest plausible daily FV intake based on previous FV interventions [28]. Based on this cut-point, 108 and 65 students were excluded as outliers at baseline and at follow-up, respectively. The analyses included 995 students (mean age at baseline 13.1 years (SD = 0.4), 51 % boys). Students completed web based questionnaires during school hours and received paper questionnaires for their parents. Parent data on occupational social class were received for 674 students (60.1 %) at baseline and 423 students (39.9 %) at follow-up. Analyses on parent-reported educational level included 567 students (57 %).

The number of teachers who were involved in implementation of the Boost curriculum at each school differed from 2 (the Boost coordinators) to all teachers (maximum number of involved teachers at year seven at intervention schools ranged from 6 to 21). We received teacher data from all 20 intervention schools in the follow-up survey (May/June 2011). All principals (*n* 20, 100 %) completed both surveys (October 2010 and July 2011). Web based questionnaires were sent to principals and teachers by email.

The Boost study adheres to all Danish ethical standards and the Declaration of Helsinki and is approved by the Danish Data Protection Agency (J.nr. 2010-54-0974). When schools were invited to participate, written information was sent to principals, parent boards and student councils at all schools explaining the implications of participating in the study. All respondents were informed that participation in the baseline and follow-up surveys was voluntary and anonymous and that all data would be handled confidentially. Parents could ask the project group to exclude their child’s baseline and follow-up questionnaires from the database by ticking a box on the front page of the parent questionnaire. Responses were treated anonymously and confidentially.

### Measures

Table 1 summarizes study measures. Outcome measure: Students’ total daily FV intake in grams at follow-up measured by the pre-coded Pro Children 24-h recall questionnaire. The questionnaire makes a valid assessment of 11-year-olds’ group mean intake [29]. In the web based questionnaires students reported how many pieces or portions of specific types of FV they consumed at three different time intervals on the previous day: before school, at school, and after school. The previous day was always a school day, as we collected data Tuesday-Friday only. Pieces and portions of FV were converted into grams based on food weights from standardized guidelines [29, 30]. Based on official Danish dietary recommendations [31] the fruit measure included maximum 100 g of 100 % natural juice regardless of the number of glasses consumed. The vegetable measure excluded potatoes.

**Table 1:** Description of outcome measure, determinants and covariates

Measure (time of assessment)	Response categories/codes	Range of continuous variables and categories of categorical variables included in analysis
*Outcome*		
**Student-reported total daily intake of FV (follow-up)**		
24-h recall questionnaire based on detailed questions on yesterday’s intake of FV on three different times of the previous school day. The fruit measure included max 100 g juice. Potatoes were excluded. Exclusion of outliers >1200 g/d	Number of portions and pieces of different fruits and vegetables	0–1200 g
*Determinants*		
**Teacher-reported dose delivered of Boost curriculum (follow-up)**		
“Which of the Boost curricular activities from the teacher manual mentioned below did you teach during the Boost intervention period September 2010–May 2011?” A similar question was asked for activities from the script for a Boost project week.	List of all Boost curricular activities to tick off (listed by number and name consistent with teacher manuals)	School-level dose: average number of Boost curricular activities delivered by teachers at each school
		Low (0 – 3.8) (reference group)
		Medium (3.9 – 6.7)
		High (≥6.8)
**Student-reported dose received of Boost curriculum (follow-up)**		
Students were asked to rate how much they liked each of the Boost curricular activities they had been exposed to during the intervention period. Each activity rated by the student counted as one activity received by the student. We added up the activities received by each student and calculated the class- and school-average.	Short description of each Boost curricular activity	School-level dose: average number of Boost curricular activities received by students at each school
		3.6–12.3 (school mean)
		Class-level dose: average number of Boost curricular activities received by students in each class
		0–13.5 (class mean)
*Covariates*		
**Student-reported total daily intake of FV (baseline)**		
(see outcome measure)	Number of portions and pieces of different fruits and vegetables	0–1200 g
*Prior “treatment” at schools*		
**Principal-reports of the school’s focus on FV prior to participation in the Boost intervention (baseline)**		
“Did your school prior to the Boost project focus on FV for example as part of project weeks or school projects?”	Yes	Yes
	No	No (reference group)
**Principal-reported FV availability at school apart from the FV delivered as part of the Boost intervention (baseline)**		
“Is it possible for students at year seven to buy the following at the school?: 1) Fruit 2) Vegetables/salad”	Yes, every day	Everyday
	Yes, most days	Most days or less (reference group)
	Some days	
	Never	
*Dose delivered of other intervention components*		
**Teacher-reported dose delivered of the pleasant eating environment component (follow-up)**		
“How often do you cut up FV when students eat FV during your lessons?”	Every time	School-level dose: proportion of teachers at each school cutting up FV every time/most times students eat FV in class
	Most times	
	Some times	
	Seldom	≤50 % (reference group)
	Never	>50 %
**Teacher-reported (only Boost coordinators) dose delivered of parental Boost newsletters (follow-up)**		
“During the school year, Boost emailed six parental newsletters for the Boost coordinators to post on the schools’ website for parents. How many of these were posted?”	No newsletters	School-level dose: number of posted newsletters at each school
	One newsletter	
	Two newsletters	0–3 newsletters (reference group)
	Three newsletters	4–6 newsletters
	Four newsletters	
	Five newsletters	
	Six newsletters	
*Socio-demographic factors*		
**Student-reported gender (baseline)**		
“Are you a boy or a girl?”	Boy	Boy
	Girl	Girl (reference group)
**Student-reported family occupational social class (baseline)**		
“Mother’s/father’s job title” (written answer)	I High	High: I and II
“Mother’s/father’s workplace” (written answer)	II	Medium: III and IV (reference group)
Based on job title and place of work of the mother and father, each parent was coded into one of five occupation social classes or some additional groups using standardized coding principles. Family occupational social class was based on the highest ranking parent. Students who did not provide sufficient information to code parents into occupational social classes or additional groups were excluded from the analyses.	III	Low: V and 7
	IIII	Unclassifiable: 6
	V Low	
	6 Has a job, but information unclassifiable	
	7 Social Welfare benefits	
**Parent-reported family educational level (baseline)**		
“Which school education do you have?”	Enrolled in education	High education: f
“Which vocational education do you have?” (If you have more than one, please tick off the highest level of education)	Primary school	Medium high education: e
	Manual education	Low education/none: a-d (reference group)
	Low theoretical education	
Based on completed education, mothers and fathers were categorized into one of five educational categories using national coding principles. Family educational level was based on the highest ranking parent. Unclassifiable parents were excluded.	Medium high theoretical education	
	High theoretical education	

Determinants: *Dose delivered* of the Boost curriculum at each school (school-level) was assessed by calculating the average number of Boost curricular activities reported to be delivered by teachers at each school during the intervention period (it was not possible to estimate the exact number of optional activities implemented). As teachers within the same school were responsible for teaching different year seven classes, teachers’ reports of delivered curricular activities did not necessarily represent one single class. Therefore it was not possible to identify the dose delivered in each participating year seven class (class-level) from the teacher questionnaire data. *Dose received* of the Boost curriculum at the school- and class-level, respectively, was calculated as the average number of Boost curricular activities received by students at each school and in each class.

Covariates: 1) Students’ self-reported total daily FV intake in grams at baseline; *Prior ‘treatment’* (principal data): 2) Schools’ focus on FV prior to the school’s participation in the intervention; 3) Students’ access to FV in school besides the free Boost FV; We controlled for *dose delivered of other intervention components* to isolate the effect of the curricular activities on students’ FV intake (teacher data): 4) Number of Boost parental newsletters delivered by Boost coordinators. At six schools, teacher data on number of newsletters uploaded were missing and substituted by parent data on number of newsletters received; 5) Dose delivered of a pleasant eating environment measured by how often the FV were cut up in appealing snacks; *Socio-demographic factors* (student- and parent data): 6) Gender; 7) Students’ information on their parents’ job title and workplace was used to code parents into occupational social classes based on standardized coding principles [32, 33]. We used student data instead of parent data for occupational social class to avoid exclusion of too many students; 8) Parents’ information on their educational background was coded into educational levels according to national coding principles [34]. Family occupational social class and family educational level were determined by the highest ranking parent.

### Statistical analyses

The association between curriculum dose and students’ daily FV intake at the end of intervention was analysed by multi-level analyses including school-, class- and individual-level. The association was adjusted for FV intake at baseline, prior ‘treatment’, and dose delivered of other intervention components. Potential moderation was examined by 1) including interaction terms between dose and gender, and dose and family occupational social class, and dose and family educational level in three separate analyses and 2) analyses stratified by the potential moderators. We excluded control schools from the analyses as the Boost curriculum was delivered at intervention schools only.

In sensitivity analyses we examined the implications of changing cut-points for outliers using >1000 and >1500 g FV daily, respectively, and of different cut-points for categorizing level of curriculum dose delivered.

In attrition analyses we tested for differences in baseline measures between students with and without a follow-up assessment.

We found a weak collinearity between dose delivered of other intervention components and curriculum dose (Spearman’s correlation coefficients <0.40).

We tested for linearity between FV intake at follow-up and curriculum dose, and FV intake at baseline and curriculum dose by 1) visual inspection of scatter plots, 2) creating various categorical variables to test if an upward or downward curve existed, and 3) square root term included in the model. Dose delivered was included in the analysis as a categorical variable including low, medium and high dose as we identified an upward curve for dose delivered against FV intake. Dose received was included in the analysis as a continuous variable as a linear tendency was seen for dose received and FV intake.

Model assumptions were evaluated using visual inspection of residual plots and QQ-plots, and Kolmogorov-Smirnov test for normality. FV intake at follow-up had a skewed distribution, but various transformations including log, square root and rank transformation of FV intake at follow-up did not normalize the distribution. Rank-transformation of the outcome measure showed similar trends in *P*-values and estimates as seen in the non-transformed analysis. We conducted analyses of the non-transformed outcome measure using the statistical software package SAS version 9.3 and chose a priori a 5 % significance level. Missing data were excluded from the analyses.

## Results

Attrition analysis: Students without follow-up data were more likely to be boys (60.8 % versus 50.7 %, *P* = 0.03), have parents with medium (45 % versus 35 %, *P* = 0.05) or low education (46.7 % versus 44.2 %, *P* = 0.05) and to have a smaller daily FV intake (mean/median 352/270 g versus 395/340 g, *P* = 0.14).

Table 2 shows that for students at intervention schools, the mean FV intake at baseline was 387 g (SD = 290.2; median = 325)/day. At follow-up, the mean intake was 407.1 g (SD = 289.3; median = 350)/day. Seven intervention schools delivered a low dose of curricular activities to students; six schools delivered a medium dose; and seven schools a high dose. The average dose delivered ranged from 0 (2 schools) to 10.7 (1 school) activities. The average dose received in classes ranged from 3.6 (2 schools) to 12.3 (1 school). The majority of schools (70 %) had focused on FV prior to participation in the Boost intervention and had FV available for purchase (65 %). In 11 schools, teachers had uploaded at least four of the six parental Boost newsletters. In 10 schools, more than 50 % of the teachers cut up the Boost FV every time or most times they distributed FV in their classes.

**Table 2:** Characteristics of the study population and distribution of daily FV intake (*n* 995)

**Individual-level characteristics**	***n*** **(%)**	**FV intake at baseline, g Mean, median (SD)**	**FV intake at follow-up, g Mean, median (SD)**	**Missing (** ***n*** **)**
*Students’ daily FV intake*	995	395, 340 (291.7)	407, 350 (289.3)	
*Gender*				
Boys	507 (51.0)	382, 300 (305.1)	380.8, 330 (280.4)	
Girls	488 (49.1)	408, 360 (276.4)	434.4, 380 (296.0)	
*Family occupational social class*				55
High	259 (27.6)	449, 398 (306.1)	452, 400 (307.5)	
Medium	265 (28.2)	377, 315 (285.8)	416, 375 (293.8)	
Low	229 (24.4)	376, 313 (292.5)	378, 328 (268.7)	
Unclassifiable	187 (19.9)	356, 300 (293.6)	344, 300 (269.6)	
*Family educational level*				428
High	118 (20.8)	465, 400 (291.1)	455, 395 (310.9)	
Medium high	200 (35.3)	423, 400 (287.5)	448, 400 (294.4)	
Low/none	249 (43.9)	382, 330 (288.0)	413, 350 (287.1)	
**School- and class-level characteristics (data source)**	***n***_**students**_ **(%) or mean (range)**		***n***_**schools**_ **(%)**	
*Curriculum dose delivered at school-level (teacher-reported)*				
Low	363 (36.5)		7 (35.0)	
Medium	256 (25.7)		6 (30.0)	
High	376 (37.8)		7 (35.0)	
*Curriculum dose received at school-level (student-reported)*	Mean 6.9 (range: 3.6–12.3)			
*Curriculum dose received at class-level (student-reported)*	Mean 6.9 (range: 0–13.5)			
*School’s focus on FV prior to the Boost intervention*				
Yes	649 (65.2)		14 (70.0)	
No	346 (34.8)		6 (30.0)	
*FV availability at school apart from the Boost distribution*				
Every day	712 (71.6)		13 (65.0)	
Most days or less	283 (28.4)		7 (35.0)	
*Newsletters: dose delivered at school-level*				
2-3	448 (45.0)		9 (45.0)	
4-6	547 (55.0)		11 (55.0)	
*Pleasant eating environment: dose delivered at school-level*				
≤50 %	461 (46.3)		10 (50.0)	
>50 %	534 (53.7)		10 (50.0)	

### Teacher-reported curriculum dose delivered

The association between average curriculum dose delivered at the school-level and students’ FV intake at follow-up was insignificant (Table 3). However analysis indicated a non-graded association (*P* = 0.75): Students at schools where teachers on average had delivered a high curriculum dose ate 31 g (CI: −85.81, 148.61) more FV at follow-up compared to students at schools with low dose delivered. Students at schools with medium dose delivered ate 51 g (CI: −77.88, 179.28) more FV than students at schools with low dose. Analysis with interaction terms showed moderation by family occupational social class (*P* = 0.04), but not family educational level and gender. Stratified analyses by gender, family occupational social class and family educational level showed no significant associations (Table 3, results for family educational level not shown).

**Table 3:** Association between *teacher-reported* curriculum dose delivered at school-level and students’ FV intake (*n* 995)

Variable	Estimate (g/d)	CI95%	P**
***Analysis of all students*** a, b*			
Curriculum dose delivered at *school-level*			0.75
Low dose	*Ref.*		
Medium dose	31	−85.81, 148.61	
High dose	51	−77.88, 179.28	
Interaction term dose* gender	.		0.18
Interaction term dose* family occupational social class	.		**0.04**
***Analysis stratified by gender*** a*			
Curriculum dose delivered at *school-level*			
*Girls*			
Low dose	*Ref.*		.
Medium dose	−29	−172.66, 115.46	0.70
High dose	−2	−135.57, 131.77	0.97
*Boys*			
Low dose	*Ref.*		.
Medium dose	101	−34.64, 236.24	0.16
High dose	65	−57.9, 187.1	0.32
***Analysis stratified by family occupational social class*** **a** *			
Curriculum dose delivered at *school-level*			
*High family occupational social class*			
Low dose	*Ref.*		.
Medium dose	138	−52.42, 328.22	0.18
High dose	35	−150.51, 221.11	0.72
*Medium family occupational social class*			
Low dose	*Ref.*		.
Medium dose	−13	−186.66, 159.86	0.88
High dose	41	−114.72, 196.92	0.61
*Low family occupational social class*			
Low dose	*Ref.*		.
Medium dose	−34	−217.76, 149.16	0.71
High dose	−17	−159.97, 126.97	0.82

* a Adjusted for differences in FV intake at baseline, schools’ prior “treatment” and dose delivered of other intervention components, b also adjusted for gender and family occupational social class **Significant associations in bold (*P* < 0.05)

### Student-reported curriculum dose received

There was no association between average dose received at the school-level and students’ FV intake at follow-up (*P* = 0.65). Analysis with interaction terms for family occupational social class was significant (*P* = 0.01), but insignificant for family educational level and gender. Stratified analyses by gender, family occupational social class and family educational level showed no significant associations (Table 4, results for family educational level not shown).

**Table 4:** Association between *student-reported* curriculum dose received at school- and class-level and students’ FV intake (*n* 995)

Variable	Mean increase in intake (g/d)	CI95%	P**
***Analysis of all students*** a, b*			
Curriculum dose received at *school-level*	5	−15.109, 24.483	0.65
Interaction term dose* gender	.		0.84
Interaction term dose* family occupational social class	.		**0.01**
***Analysis stratified by gender*** a*			**.**
Curriculum dose received at *school-level*			
*Girls*	6	−16.818, 28.262	0.63
*Boys*	1	−21.54, 23.148	0.95
***Analysis stratified by family occupational social class*** a*			
Curriculum dose received at *school-level*			
*High family occupational social class*	17	−9.957, 44.375	0.23
*Medium family occupational social class*	−11	−38.002, 15.388	0.42
*Low family occupational social class*	−3	−30.45, 25.606	0.86
Analysis of all students a, b*			
Curriculum dose received at *class-level*	10	0.055, 20.329	**0.05**
Interaction term dose* gender	.		0.27
Interaction term dose* family occupational social class	.		0.57
***Analysis stratified by gender*** a*			
Curriculum dose received at *class-level*			
*Girls*	4	−9.828, 16.828	0.61
*Boys*	15	2.844, 26.756	**0.02**
***Analysis stratified by occupational social class*** a*			
Curriculum dose received at *class-level*			
*High family occupational social class*	8	−10.332, 5.732	0.41
*Medium family occupational social class*	13	−3.068, 29.468	0.12
*Low family occupational social class*	4	−15.984, 4.784	0.67

* a Adjusted for differences in FV intake at baseline, schools’ prior “treatment” and dose delivered of other intervention components, b also adjusted for gender and family occupational social class **Significant associations in bold (*P* < 0.05)

We found a dose—response association between the average curriculum dose received by students at class-level and daily FV intake (*P* = 0.05). Students ate 10 g (CI: −0.09, 20.29) more FV per day for each extra curricular activity received at the class-level. We found no significant interaction terms. In stratified analysis, average curriculum dose received by students at the class-level was significantly associated with boys’ intake (15 g FV, CI: 2.84, 26.76) but not girls’ intake (4 g FV, CI: −9.828, 16.828). Stratified analyses by family occupational social class and family educational level showed no significant associations (Table 4, results for family educational level not shown).

Findings were robust to changes in cut-points for curriculum dose and outliers.

In multi-level models including dose received as outcome, the between-class variation in dose received was larger than the between-school variation (Intraclass Correlations (ICC)—20.1 % versus 8.7 %).

## Discussion

Our study has four key findings. Firstly, the results show no significant association between the average dose of curricular activities delivered by teachers at each school and students’ FV intake at the end of intervention. Similarly we found no association between the average dose of curricular activities received by students at each school and FV intake. Secondly, we found a significant dose—response association between the average dose received by students in each class and FV intake. Students’ FV intake increased by increasing intervention dose received as hypothesized. Thirdly, the association between dose delivered at the school-level and FV intake differed by family occupational social class but not by gender. Fourthly, in stratified analyses dose received at class-level was significantly associated with FV intake among boys but not girls.

Similar to our study Bere et al. (2006) found no association between teacher-reported curriculum dose delivered at school-level and students’ FV intake, while three studies [12, 17, 35] reported a positive association. These inconsistent results might be explained by differences in teachers’ implementation level and survey response rates. In all the previously mentioned studies including the one by Bere et al. (2006), the curriculum dose was reported to be high or good, whereas the dose delivered of the Boost curriculum was low compared to the intended maximum number of compulsory activities (16 activities). Compared to the other studies, there was a low response rate among teachers in the Boost intervention. The teacher survey was administered to all teachers at year seven in the Boost intervention, but at some schools only a few teachers answered the teacher survey. Dose delivered by these teachers might not be representative for the dose delivered by teachers in other classes within the school which questions the validity of the teacher-reported dose delivered. However the low response rate at some schools may reflect that only few teachers were involved in implementation of the Boost curriculum and therefore being the only ones who found it relevant to complete the survey.

Although the literature suggests that girls in general respond better to school-based interventions addressing energy balance behaviour than boys [21], our results indicate that the dose of a curriculum is more important for changing boys’ FV intake. This does not necessarily mean that curricular activities are not important for girls’ FV intake but there might be a threshold value above which an extra curriculum dose has no impact on girls’ intake. Furthermore it might be difficult to increase girls’ FV intake as girls FV intake is already high. Our study did not find that the association between dose of curricular activities and FV intake differed by family occupational social class or family educational level. Previous studies on socioeconomically differential effects of behaviour change interventions show inconsistent results [4, 21, 36]. Generally, these studies included children and adolescents younger and older than the 13-year-old students in the Boost intervention. Furthermore these studies examined interventions not only targeting FV intake and only some interventions included curricular activities. One review studying interventions targeting energy balance-related behavior, including FV intake, among 4–18-year-olds did not find moderating effect of SEP in interventions targeting FV intake [21]. However sub-groups with low baseline level of FV intake responded better to FV interventions. Another review finds inconsistent results for interventions involving curricular activities targeting healthy diet among 13–18-year-olds [4].

Our results indicate in agreement with Durlak and DuPre (2008) that the assessment of the average intervention dose at the school-level is too crude as it ignores the fact that dose delivered and received may differ by classes within the same school. The Boost curricular component was to be implemented by teachers in each of the year seven classes, which might have resulted in different number of curricular activities delivered across classes within the same school. Furthermore, the importance of measuring intervention dose at the class-level is supported by the ICCs showing greater variation between classes in curriculum dose received than between schools. As teachers within the same school were responsible for teaching different year seven classes, it was unfortunately not possible to identify the dose delivered in each class (class-level) from the teacher questionnaire. Originally we intended to collect information on dose delivered by teachers in each participating class by teacher log books. However response rate for these log books was low.

It might influence the results whether students were exposed to curricular activities during the entire intervention period as intended and whether the students were presented for highly adapted activities. The teacher questionnaire included overall information on time of implementation and degree of adaptation of the Boost curriculum (not for each activity and class). However it was not possible to include this information in the analyses due to a low number of teachers completing the questionnaire at some schools.

Results from stratified analyses should be interpreted with caution due to small sample sizes which may be part of the reason why we find no differential effect by SEP [21]. Particularly educational subgroups included few observations due to low response rate among parents.

The non-graded association between dose delivered at school-level and students’ FV intake might be explained by the fact that a high dose delivered does not necessarily reflect a high quality delivery. Quality and level of fidelity (whether teachers have adhered to teacher manuals) seem to be important for the outcome [14]. A high dose delivered may be at the expense of high fidelity.

To prevent social desirability bias among students, the Boost project group introduced the questionnaire by emphasizing that it was not an exam and that there were no right or wrong answers. Students were encouraged to answer as honestly as possible. Teachers may have over-reported their implementation of the compulsory curriculum to please the researchers who have provided them with FV and teaching material for free. In a qualitative study of barriers and facilitators for implementation of the Boost curriculum, teachers felt that their position as a selected intervention school in a research project obliged them to implement the intervention [15]. Some studies [37–39] find that teachers report higher curriculum dose in surveys compared to classroom observations and post-implementation teacher interviews. Assessment of intervention dose by observational methods might have improved the implementation measures in this study. However ongoing observation was infeasible within the context of this study and there is a risk that teachers being observed act differently.

To prevent recall bias in student-reported dose received, students were presented with a short description of each Boost curricular activity instead of a title or number that could be difficult for the students to recognize. Still it might be difficult for students to remember activities implemented in the beginning of the school year when asked at the end. In the teacher survey, we listed the curricular activities by number and title consistent to the teacher manuals to prevent recall bias.

The association between curriculum dose received and FV intake may be subject to same source bias as both measures are based on students’ self-reports. Students who appreciate the Boost intervention might over-report curriculum dose received. However we minimized this risk by calculating the dose received by students on average in each class and school instead of using dose received at the individual level.

Strengths of this study include a comprehensive analysis of the role of both dose delivered and dose received, the multiple data sources, high response rates among students and principals, and the use of a validated 24-h recall questionnaire. The 24-h recall gives a valid assessment of group-level mean intake [29]. However single 24-h recalls cannot give an accurate representation of usual individual dietary intake due to intra-individual day-to-day variations in diet. To our knowledge, this is the first study to examine the role of curriculum dose reported by students at the school- and class-level for behavioural change.

Significantly more boys and students with parents with low or medium education were lost to follow-up in this study. As students’ FV intake shows a social gradient by parental educational level, implications of this finding might be that we over-estimate the relationship between dose and intake among students with parents with high educational level. Also, there is a risk that boys lost to follow-up have a lower intake than boys who stay in the study.

Future process evaluations of interventions should aim to assess intervention dose delivered and received at the class-level. Ways to increase teachers’ response rate should be explored in order to get more valid assessment of dose delivered. For example by emphasizing the importance of all teachers answering the questionnaires independent of their involvement in implementation. Otherwise, student-reported dose received aggregated to the class-level is probably the most valid measure. We identified a dose–response association with increasing FV intake by higher dose received by students at class-level. Future studies should examine whether a minimum curriculum dose is needed or if a threshold value exists above which extra activities will not contribute to further increase in adolescents’ FV intake. Future studies should examine the effect of curricular components on the specific determinants of adolescents’ FV intake they have been tailored to in order to identify mediators for behavioural change.

Our results indicate that it was possible to influence the FV intake in an adolescent subgroup with low FV intake at baseline (boys) by the educational strategy in the Boost intervention. The explorative subgroup analyses should be replicated in a larger sample of adolescents to learn more about the relationship between curriculum dose and FV intake in subgroups with different baseline levels of FV intake; for example to examine if a threshold value for dose received among adolescent girls exists as girls are found to have better dietary knowledge than boys [40, 41]. Answers to these questions will be crucial for school-based interventions with the potential of reaching all children and adolescents including those who do not already have a high FV intake.

This study indicates that receiving curricular activities focusing on FV has an influence on students’ FV intake. Curricular activities seem to be an important component in future school-based interventions targeting adolescents’ FV intake. To ensure a sustained focus on nutrition and health in schools, considerations should be done in terms of integrating these topics permanently in the regular curriculum.

## Conclusions

This study shows a dose—response association between the average student-reported curriculum dose received at the class-level and students’ FV intake. The average dose delivered and received at the school-level was not associated with students’ intake. Future studies should assess intervention dose at the class-level. The results indicate that curriculum dose received has different effects on FV intake among boys and girls. Future studies should explore such gender differences in larger samples to guide the planning of school-based curricular interventions with regards to the optimal number and types of curricular activities required to promote behavioural change in subgroups with low FV intake at baseline.

## Abbreviations

FV: Fruit and vegetable(s)
SEP: Socioeconomic position
